# Antrodin C Inhibits Epithelial-to-Mesenchymal Transition and Metastasis of Breast Cancer Cells *via* Suppression of Smad2/3 and β-Catenin Signaling Pathways

**DOI:** 10.1371/journal.pone.0117111

**Published:** 2015-02-06

**Authors:** K. J. Senthil Kumar, M. Gokila Vani, Pin-Ju Chueh, Jeng-Leun Mau, Sheng-Yang Wang

**Affiliations:** 1 Department of Forestry, National Chung Hsing University, Taichung, Taiwan; 2 Institute of Biomedical Sciences, National Chung Hsing University, Taichung, Taiwan; 3 Department of Biotechnology, Asia University, Taichung, Taiwan; 4 Department of Food Science and Biotechnology, National Chung Hsing University, Taichung, Taiwan; 5 National Chung Hsing University/University of California at Davis, Plant and Food Biotechnology Center, National Chung Hsing University, Taichung, Taiwan; 6 Agricultural Biotechnology Research Center, Academia Sinica, Taipei, Taiwan; The University of Hong Kong, CHINA

## Abstract

Epithelial-to-mesenchymal transition (EMT) is a crucial event involved metastasis of certain tumors. Thus, identifying chemical agents that can block EMT is highly warranted for the development of anti-cancer chemoprevention/chemotherapies. In this study, we found that Antrodin C (ADC), a maleimide derivative isolated from *Antrodia cinnamomea* health food product inhibits TGF-β1-induced EMT and breast cancer cell metastasis *in vitro*. Pretreatment of MCF-7 cells with ADC significantly blocked TGF-β1-induced phenotypic changes and actin cytoskeleton remodeling. In addition, ADC was able to up-regulate epithelial markers such as E-cadherin and occludin, whereas mesenchymal markers including N-cadherin and vimentin were significantly inhibited, possibly through the modulation of transcriptional regulators Smad/Smad3. ADC blocked TGF-β1-induced migration and invasion of MCF-7 cells through the down-regulation of matrix-metalloproteinases (MMP-2, -9) and urokinase plasminogen activator (uPA). The inhibition of MMPs and uPA activity by ADC was reasoned by suppression of its corresponding transcription factor β-catenin. Taken together, our data suggested that ADC attenuates the TGF-β1-induced EMT, migration and invasion of human breast carcinoma through the suppression of Smad2/3 and β-catenin signaling pathways.

## Introduction

Breast cancer is the most common malignancy and ranks number one as a cause of cancer-related death in women worldwide. Every year, above 1.3 million women are diagnosed with breast cancer and nearly 450,000 women die from it [1]. Death from breast cancer primarily attributes to cancer metastasis, a process that cancer cells invade surrounding tissues and migrate to distal organs including lung, liver, brain, bone, and lymph nodes followed by formation of secondary tumors [2]. Several steps are involved in cancer cell metastasis, including epithelial-mesenchymal transition (EMT), matrix degradation, invasion, intravasation, extravasation, adhesion, and mesenchymal-epithelial transition (MET) [3]. Among them, EMT plays a crucial role in cancer cell metastasis, as it is the first step in the migration of tumor cells [3,4]. The primary event of an EMT is the loss of epithelial markers such as E-cadherin, occluding, claudin, desmoplakin, cytokeratin-8, -9, -18, and mucin-1, followed by increased expression of mesenchymal markers such as, N-cadherin, vimentin, vitronectin, fibronectin, FSP1, and smooth-muscle actin and rearrangement of the cytoskeleton [5].

Numbers of previous studies have shown that EMT can be regulated by several factors including hypoxia, tumor-stromal cell interactions, and growth factors, such as hepatocyte growth factor (HGF), epidermal growth factor (EGF), fibroblast growth factor (FGF), insulin-like growth factor (IGF), and transforming growth factor-β (TGF-β) [6]. Among them, TGF-β, a multifunctional cytokine plays important role in the metastatic spread of breast cancer cells, through induction of migration, invasion, and EMT [7,8]. Upon the binding of TGF-β1 receptors by its ligand, Serine/Threonine kinases is activated and induces phosphorylation of Smad2/Smad3 (receptor-activated Smads), leading to the formation of heteromeric complexes with Smad4 (common-mediator Smad). These heteromeric Smad complexes then translocate into the nucleus, where numbers of EMT regulatory genes including Snail, Slug, TWIST, and Sip1 are transcribed [9,10], resulting in repression of expression of epithelial markers and induction of mesenchymal transition [9].

A strong association has been established between altered expression of the matrix metalloproteinases (MMPs) and cancer progression. MMP are important endopeptideases, acting on the degradation of extracellular matrix proteins and, in turn, promoting tumor cell metastasis [11]. Interestingly, TGF-β1 is reported to induce MMP-2 and MMP-9 activity in a variety of human cancers [12], including breast cancer [13]. Similarly, uPA, also matrix protease, plays a critical role in breast cancer metastasis by cleaving extracellular matrix, and modulating cell adhesion and migration [14]. Moreover, previous studies have identified that β-catenin, a transcription factor, involves in breast cancer metastasis via induction of MMP2, MMP-9, and UPA genes [15]. Cellular β-catenin level are tightly regulated by a constitutively active enzyme known as glycogen synthase kinase-3 β (GSK3β), which phosphorylates and thereby targets cytosolic β-catenin for proteasomal degradation [16]. In contrast, stabilized (non-phosphorylated) β-catenin translocates into the nucleus and binds to the T-cell factor (TCF)/lymphoid enhancer factor (LEF), where it activates several downstream target genes including MT-MMP-1, MMP-2, MMP-9, uPA, and EMT marker genes [17–19]. Recently, various growth factors including TGF-β1, have been demonstrated to activate β-catenin signaling pathway through GSK3β inhibition, which cooperates with Smad2/Smad3 signaling to induce transcription of EMT marker genes [19]. Accumulating evidence also indicate that activation of both TGF-β1 and β-catenin signaling pathways have significant impact on breast cancer metastasis and poor diagnosis [20]. Thus, inhibition of EMT and matrix degradation may provide therapeutic potential for improving the prognosis of breast cancer patients.

Natural products offer promising options for cancer prevention, reflecting their anti-cancer activity. *Antrodiacinnamomea* is a medicinal mushroom endemic to Taiwan. This unique fungus grows inner hearwood of age old tree *Cinnamomum kanehirai* Hayata (Lauraceae). In traditional Chinese medicine (TCM), this mushroom is used for treating food poisoning, drug intoxication, diarrhea, abdominal pain, hypertension, skin irritation, inflammation, and even cancer [21]. Accumulating scientific evidences also indicate that this mushroom exerts an array of biological activities including, hepatoprotectiion, neuroprotection, anti-oxidant, anti-inflammation, anti-hypertensive, anti-hyperlipedemic, anti-cancer and anti-metastatic effects [22]. In past two-decades, this mushroom received much attention by pharmaceutical and nutraceutical industries because of its bioactive components including, triterpinoids, polysaccharides, benzenoids, benzoquinone derivatives, and maleic/succinic acid derivatives [22]. Previously, Nakamura and his co-workers isolated 5 new maleic and succinic acid derivatives from the mycelia of *Antrodiacinnamomea*, and among them, the malemide derivatives (Antrodin B) and Antrodin C (inhibit growth of Lewis lung carcinoma cells *in vitro* [23]. The pyrrolidione, ADC also showed a strong inhibition against lipopolysaccharide (LPS)-induced pro-inflammatory cytokines and nitric oxide production in macrophage cells [24,25]. However, the anti-metastatic potential of malemide derivatives is largely unknown.

**Fig 1:**
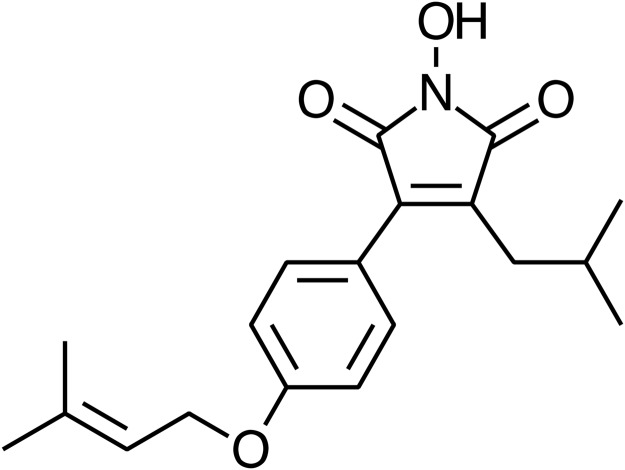
Chemical structure of Antrodin C (ADC). Synonym: 3-Isobutyl-4-[4-(methyl-2-butenyloxy)phenyl]-1*H*-pyrrole-2,5-dione; Molecular formula: C_19_H_23_NO_4_; Molecular weight: 329.39.

In the present study, we used MCF-7, an epithelial line derived from metastatic site for mammary gland, is a well-validated model to explore the ability of ADC (Fig. 1) in inhibiting the TGF-β1-induced phenotypic changes associated with EMT. Also, we investigated TGF-β1/β-catenin-mediated extracellular matrix degradation, migration, and invasion of breast cancer cells. Our results demonstrate that ADC inhibits TGF-β1-induced changes in EMT markers *via* down-regulation of Smad2/Smad3 signaling cascades as well as inhibits matrix degradation, migration, and invasion of breast cancer cells through the inhibition of β-catenin signaling pathway. This is the first report demonstrating the anti-metastatic ability of ADC, an active constituent from mycelia of *A*. *cinnamomea*, despite few reports on the anti-metastatic potential of *A*. *cinnamomea*.

## Materials and Methods

### Chemicals

Dulbecco’s Modified Eagle’s medium (DMEM), fetal bovine serum (FBS), sodium pyruvate, and penicillin-streptomycin-neomycin were obtained from GIBCO BRL., (Carlsbad, CA). Antibodies against MMP-2, MMP-9, uPA, β-actin, and occluding were obtained from Santa Cruz Biotechnology Inc., (Dallas, TX). Antibodies against Slug, Snail, Twist, smad2, smad3, phos-smad2 (Ser465/467), phos-smad3(Ser423/425), β-catenin, phos-β-catenin, GSK3β, Phos-GSK3β(Ser9), E-cadherin, N-cadherin, vimentin, and MT1-MMP were purchased from Cell Signaling Technology, (Danvers, MA). All other chemicals were of the highest grade commercially available and supplied either by Merck (Darmstadt, Germany) or Sigma (St. Louis, MO). Antrodin C (ADC) was kindly provided by Taiwan Leader Biotech Corp., Taiwan.

### Cell Culture and Drug Treatment

Human breast carcinoma (MCF-7) cell line was obtained from American Type Culture Collection (ATCC, Manassas, VA) and cultured in DMEM/high glucose, 10% FBS, 1% sodium pyruvate, and 1% penicillin/streptomycin in a 37°C humidified incubator supplemented with 5% CO_2_. Cells were maintained in serum-free DMEM/high glucose for 24 h, followed by treatment with or without ADC (5–20 μM) for 2 h and 20 ng/mL of TGF-β1 (R&D Systems, Minneapolis, MN) for 0.5–48 h.

### Cell Viability Assay

The effect of ADC on MCF-7 cell viability was determined by 3-(4,5-Dimethylthiazol-2-yl)-2, 5, -diphenyltetrazolium bromide (MTT) colorimetric assay. MCF-7 cells at a density of (2× 10^4^ cells/well) were seeded in a 96-well cell culture plate, pre-incubated with ADC (5–40 μM) in the presence or absence of TGF-β1 (20 ng/mL) for 48 h. After treatment, the cells were incubated with 200 μL of 0.5 mg/mL MTT in DMEM for 2 h. Culture supernatant was then removed and re-suspended with 200 μL of dimethyl sulfoxide (DMSO) to dissolve the MTT formazan, and the absorbance was measured at 570 nm using ELISA micro-plate reader (Bio-Tek Instruments, Winooski, VT). The effect of ADC on cell viability was assessed as the percent of viable cells compared with the vehicle-treated control cells, which were arbitrarily assigned a viability of 100%. The assay was performed in triplicate at each concentration.

### Western Blot Analysis

MCF-7 cells (1 × 10^6^ cells/dish in 6-cm dish) were pre-treated with ADC (5–20 μM) for 2 h, and then incubated with TGF-β1 for the indicated time points. After treatment, the cells were detached, washed once in cold PBS, lysed by either mammalian protein extraction reagent or nuclear and cytoplasmic extraction reagent kits (Pierce Biotechnology, Rockford, IL). Protein concentrations were determined using Bio-Rad protein assay reagent (Bio-Rad Laboratories, Hercules, CA). Equal amount of protein extracts were reconstituted in sample buffer (0.062 M Tris-HCl [pH 6.8], 2% SDS, 10% glycerol and 5% β-mercaptoethanol), and the mixture was boiled for 5 min at 94°C. Denatured protein samples were separated by 8–15% SDS-PAGE, followed by transfer onto PVDF membranes overnight. The membranes were blocked with 0.1% Tween-20 in Tris-buffered saline containing 5% non-fat dry milk for 30 min at room temperature and then reacted with primary antibodies for 2 h. They were then incubated with a horseradish peroxidase-conjugated goat anti-rabbit or anti-mouse antibody for 2 h. The blots were detected by using VL Chemi-Smart 3000 (Viogene-Biotek, Sunnyvale, CA) with enhanced chemiluminescence (ECL) western blotting reagent (Millipore, Billerica, MA).

### Zymography

Intercellular MMPs activity was determined by zymography. Briefly, MCF-7 cells were cultured in 6-cm dish at a density of 5 × 10^5^ cells/dish. Then, the cells were pre-treated with or without ADC (5–20 μM) for 2 h, and then incubated with TGF-β1 for 48 h. The culture supernatant was collected, and the protein content in culture media was determined by Bio-Rad protein assay reagent using BSA as a standard. An equal amount of culture samples were subjected to 10% SDS-PAGE, which contains gelatin or casein (1 mg/mL). After electrophoresis, the gels were washed and incubated with re-nature buffer (2.5% Triton-X) for 1 h, and then incubated with developing buffer for overnight at 37°C. After overnight incubation, the bands were incubated with 0.5% Coomassie Brilliant Blue for 30 min. After appeared as white bands against a blue background with intensity in proportion to the MMPs activity. The intensities of bands were visualized by ImageQuant LAS 4000 mini (GE Healthcare Bio-Sciences AB, Sweden).

### Immunofluorescence

MCF-7 cells at a density of 2 × 10^4^ cells/well were cultured in an 8-well glass Nunc Lab-Tek chamber slide (Thermo Fisher Scientific, Waltham, MA) and pretreated with or without various concentrations of ADC (5–20 μM) for 2 h, and then incubated with or without TGF-β1 (20 ng/mL) for 1 h. After treatment, the culture media was removed and the cells were fixed in 2% paraformaldehyde for 15 minutes, permeabilized with 0.1% Triton X-100 for 10 minutes, washed and blocked with 10% FBS in PBS, and then incubated for 2 h with anti-β-catenin or anti-phospho-Smad2/3 primary antibodies in 1.5% FBS. The cells were then incubated with the fluorescein isothiocyanate (FITC) or tetramethylrhodamine (TRITC)-conjugated secondary antibody for another 1 hour in 6% BSA. Next, the nucleus were stained with 1 μg/mL 4′,6-diamidino-2-phenylindole (DAPI) for 5 minutes, washed with phosphate buffer saline (PBS), and visualized using a confocal microscope at 200 × magnification.

### Phase Contrast Microscopy

The phenotypic changes of MCF-7 cells were determined by phase contrast microscopy. MCF-7 cells were pre-treated with ADC (5–20 μM) for 2 h, and then incubated with TGF-β1 for 48 h. The morphological changes were visualized by phase contrast microscopy. The images were collected using Motic inverted microscope (Motic Instruments Inc, Canada).

### F-actin Confocal Microscopy

MCF-7 cells at a density of 2 × 10^4^ cells/well were cultured in an 8-well glass Nunc Lab-Tek chamber slide (Thermo Fisher Scientific, Waltham, MA) and pretreated with or without various concentrations of ADC (5–20 μM) for 2 h, and then incubated with or without TGF-β1 (20 ng/mL) for 24 hours. After incubation, cells were rinsed quickly in PBS for 2 times, fixed in freshly prepared 4% paraformaldehyde in PBS for 15 min at room temperature, permiabilized with Triton X-100 in PBS for 10 min, and incubated with 6% bovine serum albumin (BSA) for 1 h. Then the cells were washed with PBS for 3 times and incubated with Alexa Flour 488 Phalloidin for 15 min to stain for F-actin and then washed with PBS. Nuclear staining was performed with DAPI. Cells on slides were mounted onto cover slip with glycerol. Cells were imaged with a laser scanning microscope at 200 × magnification.

### Luciferase Reporter Assay

MCF-7 cells were transfected with control vector pCNA3 or pGL3-SBE-4-Luc or TOP-flash or FOP-flash or pCMV-β-Gal (Upstate Biotechnology, Charlottesvilla, VA) reporter gene plasmids using Lipofectamine 2000 (Invitrogen) in serum- and antibiotics-free DMEM. After 6 h, medium was changed to DMEM supplemented with antibiotics and 10% FBS, in which cells were grown for another 18 h. Then the cells were pre-treated with ADC (5–20 μM) for 2 h, and then incubated with TGF-β1 for 3 h. After incubation, cells were harvested and assayed for luciferase activity using a commercially available kit (Promega, Madison, WI). The luciferase activity was measured using luminescence ELISA micro-plate reader (Bio-Tek Instruments) and the luciferase activity was normalized with β-galactosidase activity.

### *In Vitro* Wound-Healing Repair Assay

MCF-7 cells (1 × 10^5^ cells/well) were seeded into a 12-well culture plate with silicon cell-free gap insert (ibidi GmbH, Martinsried, Germany). After monolayer formation, the insert was removed, washed with PBS, and then the cells were pre-incubated with ADC (5 and 20 μM) for 2 h, and then incubated with or without TGF-β1 for 48 h. The migrated cells were photographed (100 ×magnification) at 0 and 48 h to monitor the migration of cells into the wounded area, and the closure of the wounded area was calculated.

### *In Vitro* Invasion Assay

The matrigel invasion assay was performed in 24-well trans-well culture plates. Briefly, 10 μL (0.5 mg/mL) BD Matrigel Basement Membrane Matrix (BD Bioscience, Los Angeles, CA) was applied to 8-μm polycarbonate membrane filters, 1 × 10^5^ cells were seeded to the matrigel-coated filters in 200 μL of serum-free medium containing various concentrations of ADC (5–20 μM) in triplicate. The bottom chamber of the apparatus contained 750 μL of complete growth medium. Cells were allowed to migrate for 48 h at 37°C. After 48 h incubation, the medium was aspirated, and the non-invading cells on the top surface of the membrane were removed with a cotton swab. The invasive cells on the bottom side of the membrane were fixed in cold methanol for 15 min and washed 3 times with PBS. The cells were stained with Giemsa stain solution and then de-stained with PBS. Images were obtained using an optical microscope (200 ×magnification), and invading cells were quantified by manual counting.

### Statistical Analysis

Data are expressed as means ± SD. The significance of differences between group means were tested using Student’s *t*-test for single comparisons. *P* Values of < 0.05*, < 0.01**, and < 0.001*** were considered significant for sample versus control. A *P* value of < 0.001^ø^ was considered significant for control versus TGF-β1 alone.

## Results

### Effect of ADC on MCF-7 Cell Viability

**Fig 2:**
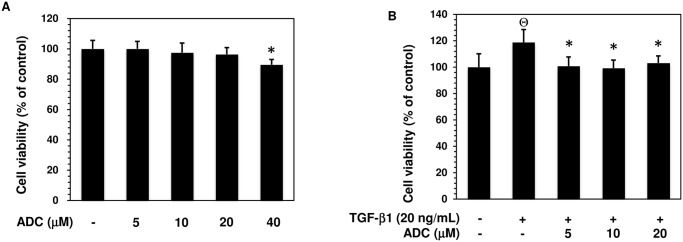
Effect of ADC on MCF-7 cell viability. (**A**) MCF-7 cells were incubated with increasing concentrations of ADC (5–40 μM) for 48 h. (**B**) Cells were pre-treated with ADC (5–20 μM) for 2 h, and then incubated with TGF-β1 for 48 h. Cell viability was determined by MTT colorimetric assay. The percentage of cell viability was cauculated by the absorption of control cells (0.1% DMSO) as 100%. The data reported as mean ± SD of three independent experiments. ^Θ^ *P*< 0.001, significant difference from control and TGF-β1 treated group. *P< 0.05 significant difference from TGF-β alone and ADC treated groups.

Prior to the investigation of anti-metastatic potential of ADC, we examined the cytotoxic effect of ADC on MCF-7 cells using MTT colorimetric assay. Results showed that treatment with ADC (5–4000B0035M) of MCF-7 cells for 48 h, cell viability was unaffected by ADC up to 20 μM. A significant reduction in cell viability was observed at concentration of 40 μM (Fig. 2A). In addition, compared with control cells, treatment with TGF-β1 (20 ng/mL) significantly increased cell number (cell proliferation), which was further inhibited significantly by ADC (Fig. 2B). Non-cytotoxic concentrations of ADC (i.e., ≤ 20 μM) was then used to evaluate its anti-metastatic potential in MCF-7 cells based on these results.

### ADC Inhibits TGF-β1-Induced Phenotypic Transition and EMT in MCF-7 Cells

**Fig 3:**
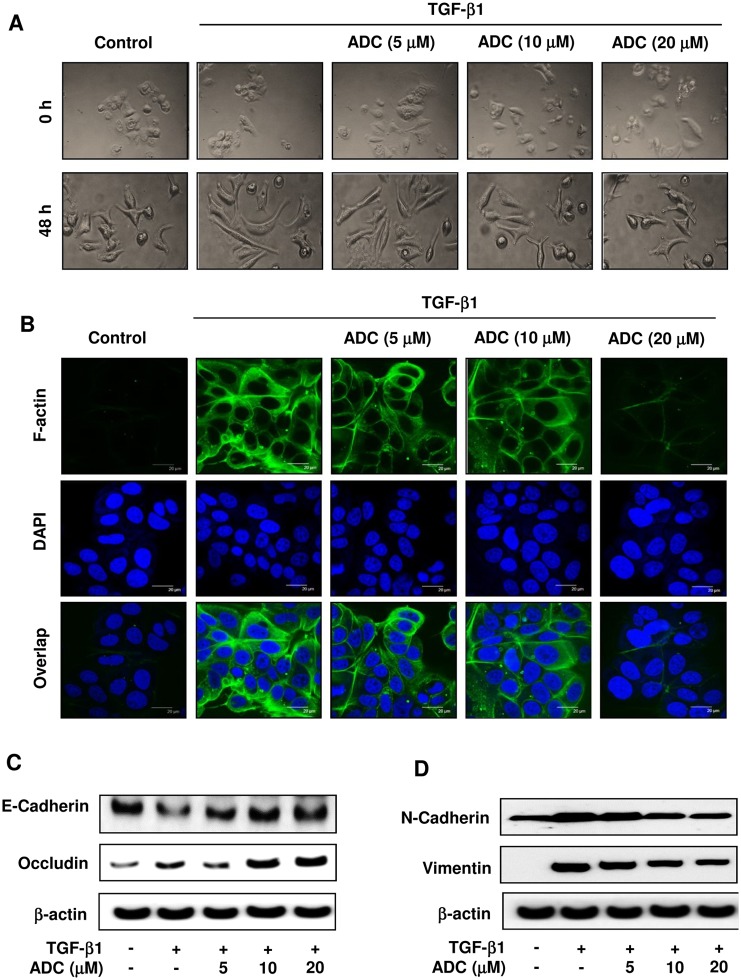
ADC blocks TGF-β1-induced EMT in breast cancer cells. MCF-7 cells were pre-treated with ADC (5–20 μM) for 2 h prior to stimulation with TGF-β1 (20 ng/mL) for 48 h. (**A**) Morphological changes especially cell scattering was examined by phase-contrast microscope. Photomicrography shown here are from one of the three independent experiments. (**B**) ADC inhibits TGF-β1-induced actin cytoskeleton reorganization in MCF-7 cells. ADC and TGF-β1 treated cells were fixed, permiabilized, and stained with FITC-phalloidin to visuvalize the F-actin cytoskeleton reorganization. The images are representative of three independent experiments. Bars, 20 μm. (**C**) Protein samples were isolated from control, ADC, and TGF-β treated cells for the detection of E-cadherin, occluding, vimentin, N-cadherin, and β-actin proteins. β-actin was used as an internal control. Western blot data presented are representative of those obtained in at least three independent experiments.

The activation of TGF-β signaling has been shown to promote phenotypic changes in various breast cancer lines [8], including MCF-7 cells [26]. To determine whether ADC affects TGF-β1-induced phenoctypic changes such as fibroblast or mesenchymal-like morphology, MCF-7 cells were pretreated with ADC for 2 h prior to stimulation with TGF-β1 for 48 h. Phase contrast microscopic analysis revealed that in control cells (absence of TGF-β1), small portion of cell morphology were somewhat mesenchymal-like, however most of the cells exhibited a pebble-like (epithelial) shape and tight cell-cell adhesion. Upon TGF-β1 stimulation, cell morphology was significantly changed into mesenchymal- or fibroblast-like structure, whereas pretreatment of cells with ADC significantly reduced the TGF-β1-induced morphological changes in MCF-7 cells in a dose-dependent manner (Fig. 3A). To ensure that ADC blocked TGF-β1-induced phenotypic change in MCF-7 cells, we also examined actin cytoskeletal reorganization or stress fibers formation that is characteristic of mesenchymal cells. MCF-7 cells were pre-treated with ADC for 2 h, and incubated with TGF-β1 for 48 h, then stained with FITC-Phalloidin, which specifically recognizes filamentous actin (F-actin) cytoskeleton. Confocal microscopy analysis revealed that F-actin was almost undetectable in control cells, upon stimulation with TGF-β1, the expression of F-actin was significantly increased showing the actin cytoskeleton organized in membrane raffles and lamellipodia regions (Fig. 3B). Interestingly, pre-treatment with ADC blocked the TGF-β1-induced F-actin formation in MCF-7 cells significantly and dose-dependently (Fig. 3B). With these lines of evidences strongly support that ADC inhibits TGF-β1-induced morphological changes in MCF-7 cells.

To further clarify whether modulation of TGF-β1-induced morphological changes in ADC treated cells resulted from dysregulation of EMT regulatory proteins, we examined the expression levels of both epithelial and mesenchymal marker proteins in MCF-7 cells. Western blot analysis showed that pre-treatment with ADC significantly inhibited the TGF-β1-induced down-regulation of epithelial markers, E-Cadherin and Occludin in MCF-7 cells (Fig. 3C). On the other hand, compared to untreated control cells, a significant increase of N-cadherin and Vimentin were observed in ADC treated cells (Fig. 3D). All these data confirmed that ADC attenuated TGF-β1-induced morphological changes and EMT in MCF-7 cells.

### ADC Represses TGF-β1-Induced Up-Regulation of Transcriptional Factors that Regulate EMT

**Fig 4:**
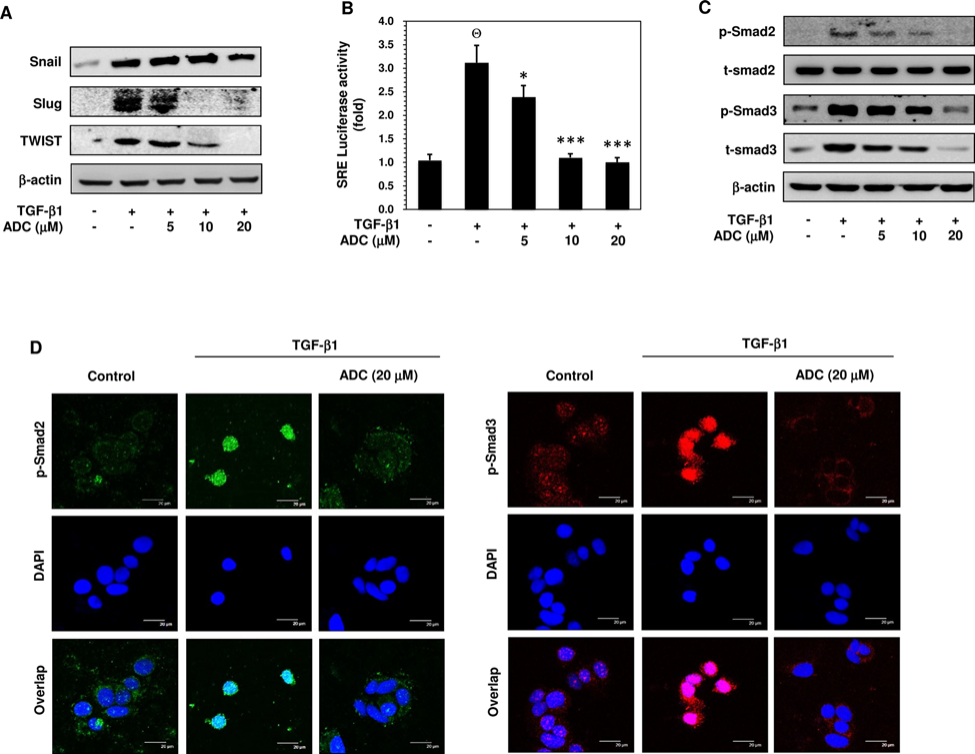
ADC inhibits TGF-β1-induced transcriptional activity of Smad2/Smad3. (**A**) MCF-7 cells were pre-treated with ADC (5–20 μM) for 2 h prior to stimulation with TGF-β1 (20 ng/mL) for 24 h. Western blot analysis was performed to examine the protein expression levels of Snail, Slug, and TWIST with specific antibodies. β-actin was used as an internal loading control. (**B**) Cells were transfected with pGL3-SBE-4-Luc reporter construct, and then pre-treated with ADC (5–20 μM) prior to stimulation with TGF-β1 for 3 h. The luciferase activity was expressed as a relative value compared to that of the untreated cells which was set to 1-fold. (**C**) Western blot was performed to measure the total and phosphorylated levels of Smad2 and Smad3 proteins. (**D**) TGF-β1-induced nuclear translocation of phosphorylated Smad2 and Smad3 were examined by immunofluorescence analysis with confocal microscope. Cells were pre-treated with ADC (20 μM) for 2 h, and then incubated with TGF-β1 for 1 h. After treatment, cells were fixed, permiabilized, and incubated with Phos-Smad2 or Phos-Smad3 primary antibodies for overnight followed by FITC and TRITC secondary antibodies, respectively for 1 h. The cellular DNA was stained with DAPI (1 μg/mL) and images were captured by confocal microscope (magnification 200). Bars, 20 μm. The data reported as mean ± SD of three independent experiments. ^Θ^ *P*< 0.001, significant difference from control and TGF-β1 alone treated group. *P< 0.05, **P< 0.01, and ***P< 0.001 were significantly different from TGF-β1 alone with the ADC treatment groups.

Recent studies have revealed that several transcription factors, including Snail, Slug, and Twist contribute to repress epithelial phenotype and activate mesenchymal phenotype in various cell lines. Therefore, we examined the effect of ADC on protein expression levels of Snail, Slug, and Twist in TGF-β1-exposed MCF-7 cells. Western blot analysis showed that treatment with TGF-β1 resulted in a marked increase of Snail, Slug, and Twist protein expression in MCF-7 cells (Fig. 4A). The TGF-β1-induced protein expressions of Slug and Twist were inhibited significantly and dose-dependently by ADC, whereas the TGF-β1-induced Snail expression was poorly inhibited by ADC (Fig. 4A).

### ADC Inhibits TGF-β1-Inducible Transcriptional Activity of Smad2/3 in MCF-7 Cells

TGF-β1 is reported to be a major secretory ligand to stimulate Smad2/Smad3 activation by acting on TGF-β-type I receptor (TβRI). The activated Smad complex binds with Smad binding element (SBE) and transcribes several EMT regulatory genes, including Snail, Slug, and Twist [9,10]. Given that ADC inhibited TGF-β1-induced Slug and Twist expression, it is of interest to examine whether ADC suppresses the transcriptional activity of Smad2/Smad3. Utilizing the SBE-harboring luciferase reporter assay, we showed that pre-treatment with ADC significantly inhibited the TGF-β1-induced luciferase activity in a dose-dependent manner (Fig. 4B). Compare to control (1-fold), TGF-β1 treatment increased luciferase activity to 3 ± 0.5-fold, whereas ADC pre-treatment reduced the luciferase activity to 2.4 ± 0.3-fold, 1.1 ± 0.2-fold, and 0.9 ± 0.1-fold at 5, 10 and 20 μM, respectively (Fig. 4B).

### ADC Inhibits TGF-β1-Induced Phosphorylation and Nuclear Translocation of Smad2 and Smad3

Phosphorylation of Smad2 and Smad3 at Ser465/467 and Ser423/425, respectively and nuclear translocation by TGF-β1 are required for transcriptional activity of Smad2 and/or Smad3. Thus, the effect of ADC and TGF-β1 on Smad2 and Smad3 phosphorylation was examined. As shown in Fig. 4C, cells pre-treated with ADC followed by TGF-β1 treatment, the ratio of phosphorylated Smad2 and Smad3 protein is comparable to the ratio in cells treated with only TGF-β1. The total Smad2 protein expression level was unaffected by neither ADC nor TGF-β1, whereas a significant increase of total Smad3 was observed in TGF-β1 treated cells, which was significantly inhibited by ADC in a dose-dependent manner (Fig. 4C). We further investigated whether ADC inhibits the nuclear translocation of Smad2 and/or Smad3 in TGF-β1-treated cells. The intracellular localization of phosphorylated Smad2 and/or Smad3 were examined by immunofluorescence using specific anti-phospho-Smad2 and anti-phospho-Smad3 antibodies. As shown in Fig. 4D, Smad2 and Smad3 were poorly phosphorylated in the absence of TGF-β1. Upon the TGF-β1 treatment, phosphorylated Smad2 and Smad3 proteins translocated into nucleus, whereas pre-treatment with ADC significantly blocked the TGF-β1-induced nuclear translocation of p-Smad2 and p-Smad3 in MCF-7 cells, confirmed by co-localization with DAPI nuclei staining.

### ADC Inhibits TGF-β1-Induced Migration and Invasion in MCF-7 Cells

**Fig 5:**
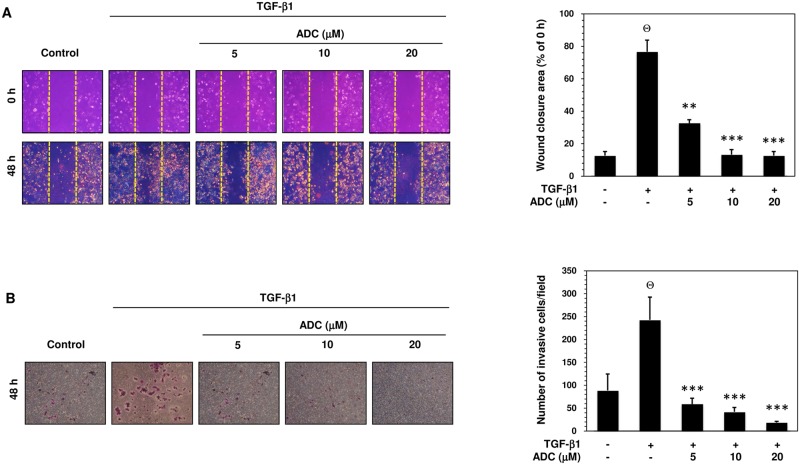
ADC inhibits TGF-β1-induced breast cancer cell migration and invasion. (A) Cell migration was determined by wound healing assay, the confluent MCF-7 monolayer was pre-treated with ADC (5–20 μM) for 2 h, cells were scratched by 200 μL pipet tips, and washed to remove the debris followed by addition of freshmedium containing 1% FBS and TGF-β1 (20 ng/mL). Cells were then incubated for 48 h. Photographs were taken at 0 h, and 48 h using inverted microscope with 10 × magnification. TGF-β1-induced cell motility was determined by measuring the area of wound closure as shown by histogram. The closure area at 48 h was compared with 0 h in the same samples. (B) For the invasion assay, the pre-treated cells were seed into the upper chamber of 24-well transwell chamber containing DMEM with 1% FBS. The lower chamber was filled with complete serum media. The cells were allowed to invade for 48 h. Invading cells were then fixed, and stained with Giemsa stain solution and counted in 5 random fields. The average invaded cells in each group was presented by histogram. The data reported as mean ± SD of three independent experiments. ^Θ^ *P*< 0.001, significant difference from control and TGF-β1 alone treated group. *P< 0.05, **P< 0.01, and ***P< 0.001 were significantly different from TGF-β1 alone with the ADC treatment groups.

TGF-β is known to stimulate malignant tumor cell migration, invasion, and metastasis via activation of MMPs [12,13]. Therefore, we further examined whether ADC also affects TGF-β1-induced MCF-7 cell migration. As shown in Fig. 5A, TGF-β1-induced cell migration was determined by measuring wound closure. The dotted lines indicate the edges of wounded area and the wound closure was photographed at 0 h and 48 h. Comparing to untreated control cells, a remarkable increase of cell migration was observed in TGF-β1 treated cells, whereas pre-treatment with ADC abrogated the TGF-β1-induced cell migration significantly and dose-dependently (Fig. 5A). The percentage of wound closure after 48 h was compared with 0 h and the values were plotted in histogram form (Fig. 5A). The percentage of wound closure in control cells were 12.7 ± 2.5%, whereas the wound closure was significantly increased to 76.7 ± 7.09%. Pre-treatment with ADC dose-dependently reduced the wound closure in to 32.7 ± 2.08%, 13.3 ± 3.06, and 12.7 ± 2.52% at 5, 10, and 20 μM (Fig. 5A). Next, trans-well invasion assay was performed to determine whether ADC is able to block TGF-β1-induced invasion. As shown in Fig. 5B, numbers of invasive cells in control group were 88.7 ± 36.12, whereas the numbers of invasive cells were markedly increased to 242.7 ± 49.86 after exposure to TGF-β1. Pre-treatment with ADC dose-dependently decreased the invasiveness of MCF-7 cells, as supported by the invaded cell numbers were decreased to 59 ± 12.77, 42 ± 9.54, and 18.7 ± 2.52 by 5, 10, and 20 μM of ADC (Fig. 5B).

### ADC Down-Regulates TGF-β1-Induced MMPs and uPA Activity in MCF-7 Cells

**Fig 6:**
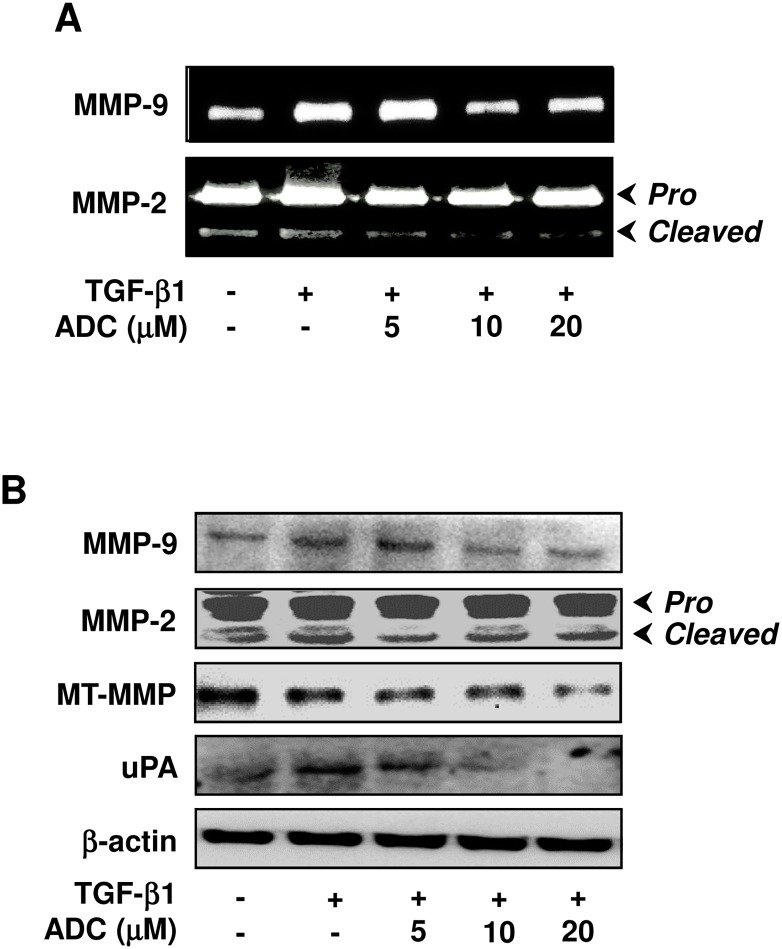
ADC down-regulates TGF-β1-induced MMPs activity in breast cancer cells. (**A**) MCF-7 cells were pre-treated with ADC (5–20 μM) for 2 h, and then stimulated with TGF-β1 (20 ng/mL) for 48 h. Gelatin zymography was performed with conditioned media collected from MCF-7 cells. (**B**) Protein expression levels of MT1-MMP, MMP-2, MMP-9, and uPA were determined by western blot analysis with specific antibodies. The house-keeping protein β-actin served as an internal loading control. Arrows denote pro- and cleaved forms of MMP-2. The data reported as mean ± SD of three independent experiments. ^Θ^ *P*< 0.001, significant difference from control and TGF-β1 alone treated group. *P< 0.05, **P< 0.01, and ***P< 0.001 were significantly different from TGF-β1 alone with the ADC treatment groups.

Next, we hypothesized that inhibition of TGF-β1-induced migration and invasion by ADC may due to the inhibition of matrix metalloproteinases activity, thus, enzyme activity of MMP-2 and MMP-9 were examined by gelatin zymography. As we expected, MMP-2 and MMP-9 enzyme activity was significantly increased following TGF-β1 stimulation. However, pre-treatment with ADC significantly as well as dose-dependently inhibited TGF-β1-induced MMP-9 and MMP-2 activity in MCF-7 cells (Fig. 6A). Interestingly, neither ADC nor TGF-β1 modulated the activity of MMP-2 pro-form, however a significant effect was observed in its matured form (Fig. 6A). To further confirm this effect, protein expression levels of metalloproteinases were examined by western blot analysis. As shown in Fig. 6B, protein expression levels of MMP-2 and MMP-9 were markedly increased by TGF-β1 and further inhibited by ADC in a dose-dependent manner. Consistent with results from zymography (Fig. 6A), ADC or TGF-β1 affected only the matured form of MMP-2 (Fig. 6B). Given that a membrane type MMP (MT-MMP) plays a crucial role in the activation of pro-MMP-2 into matured-MMP-2 in breast carcinoma cells [27], the effect of ADC on MT-MMP expression was also examined. ADC significantly inhibited the protein expression level of MT-MMP in a dose-dependent manner (Fig. 6B). Since uPA also play a major role in matrix degradation, we next examined whether ADC down-regulates uPA expression in MCF-7 cells. As shown in Fig. 6B, pre-treatment with ADC significantly inhibited the TGF-β1-induced uPA expression in dose-dependent manner. All these data suggest that ADC protects membrane matrix from degradation through the down-regulation of both gelatinolytic and plasminolytic proteins expression.

### ADC Inhibits TGF-β1-Induced Transcriptional Activation of β-catenin in MCF-7 Cells

**Fig 7:**
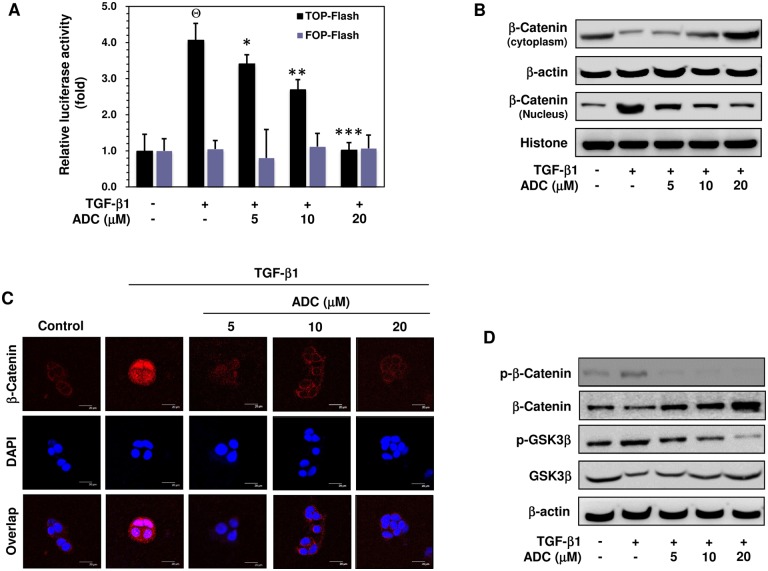
ADC suppressed TGF-β1-induced transcriptional activation of β-catenin breast cancer cells. (**A**) MCF-7 cells were co-transfected with TOP-flash or FOP-flash or pCMV-β-Gal harboring luciferase reporter construct. After transfection, cells were pre-treated with ADC (5–20 μM) for 2 h and then stimulated with TGF-β1 for 3 h. Luciferase activity was determined and normalized with β-gal activity. The histogram shows the relative luciferase activity (fold increase). (**B**) Cells were pre-treated with ADC (5–20 μM) for 2 h and then stimulated with TGF-β1 for 2 h. β-catenin expression in cytoplasam and the nucleus were determined by western blot analysis using specific cytosolic and nuclear extracts. β-actin and histone were served as an internal control for cytosolic and nuclear fractions, respectively. (**C**) The nuclear localization of β-catenin in MCF-7 cells were determined by immunofluorescence staining. MCF-7 cells were seeded in a 8-well Tek chamber and allowed to adhere for 24 h. ADC (5–20 μM) for 2 h and then stimulated with TGF-β1 for 2 h. After treatment, cells were fixed, permiabilized, and incubated with β-catenin primary antibody overnight, followed by FITC secondary antibody for 1 h. The cellular DNA was stained with DAPI (1 μg/mL) and images were captured by confocal microscope (magnification 200). (**D**) Cells were pre-incubated with ADC (5–20 μM) for 2 h, and then stimulated with TGF-β1 for 1 h. The phosphorylated and total protein expression levels of β-catenin and GSK3β were determined by western blot analysis. The data reported as mean ± SD of three independent experiments. ^Θ^ *P*< 0.001, significant difference from control and TGF-β1 alone treated group in TOP-Flash transfected groups. *P< 0.05, **P< 0.01, and ***P< 0.001 were significantly different from TGF-β1 alone with the ADC treatment groups in TOP-Flash transfected groups.

Recent progress has been made to identify the upstream signaling molecule of MT-MMP, MMP-2, MMP-9, and uPA as β-catenin [17–19]. Therefore, we next determined whether ADC inhibits TGF-β1-induced transcriptional activation of β-catenin. MCF-7 cells were pre-incubated with ADC (5–20 μM) for 2 h, and then exposed to TGF-β1 for 2 h. The TGF-β1-induced activation of TCF/LEF-dependent gene transcription was assayed using β-catenin sensitive TOP-flash and random FOP-flash luciferase assay. As shown in Fig. 7A, the luciferase activity in MCF-7 cells transfected with TOP-flash vector was markedly increased to 4.07 ± 1.0-fold in response to TGF-β1 stimulation, whereas pretreatment with ADC significantly as well dose-dependently decreased TGF-β1-induced TOP-flash activity to 3.4 ± 0.8-fold, 2.6 ± 1.1-fold, and 1.0 ± 1.0-fold at 5, 10, and 20 μM, respectively (Fig. 7A). However, cells transfected with the negative control FOP-flash reporter vector was unaffected by either TGF-β1 or ADC (Fig. 7A).

### ADC Inhibits TGF-β1-Induced Nuclear Translocation of β-catenin in MCF-7 Cells

It has been implicated that TGF-β-1 triggers β-catenin nuclear translocation, where it can associate with transcription factors such as TCF/LEF to activate expression of their target genes. Thus, we determined the effect of ADC on the cellular location of β-catenin protein using cell fractionation. Western blot analysis revealed that treatment of MCF-7 cells with TGF-β1 markedly increased β-catenin level in the nucleus, whereas pretreatment with ADC resulted in reduction of β-catenin level in the nucleus in a dose-dependent manner (Fig. 7B). This change is correlated with dose-dependent increase in cytosolic β-catenin (Fig. 7B). This observation was further confirmed by immunofluorescence analysis, demonstrating an increased staining of nuclear β-catenin in response to TGF-β1stimulation that was further reduced by ADC pretreatments (Fig. 7C). Concurrently, the cytosolic β-catenin was significantly increased in the ADC-exposed cells as supported by immunofluorescence staining (Fig. 7C). These data suggest that pretreatment with ADC significantly inhibited the TGF-β1-induced nuclear translocation of β-catenin in MCF-7 cells.

### ADC Inhibits TGF-β1-Induced Phosphorylation of β-catenin and GSK3β in MCF-7 Cells

β-catenin expression and localization are tightly regulated by a cytoplasmic multiprotein complex containing GSK3β, which in its unphosphorylated form targets unbound cytoplasmic β-catenin for intracellular breakdown. TGF-β1 is reported to induce β-catenin activity through the inhibition of GSK3β. Consistent with this connection, stimulation of MCF-7 cells with TGF-β1 induced a profound increase of GSK3β phosphorylation and a decrease of total GSK3β level in the cytoplasm, while β-catenin phosphorylation was enhanced significantly (Fig. 7D). Furthermore, pretreatment with ADC significantly inhibited the TGF-β1-induced phosphorylation of GSK3β and increased total GSK3β in cytoplasm, which is highly correlated with increased β-catenin level in the cytoplasm (Fig. 7D).

## Discussion

**Fig 8:**
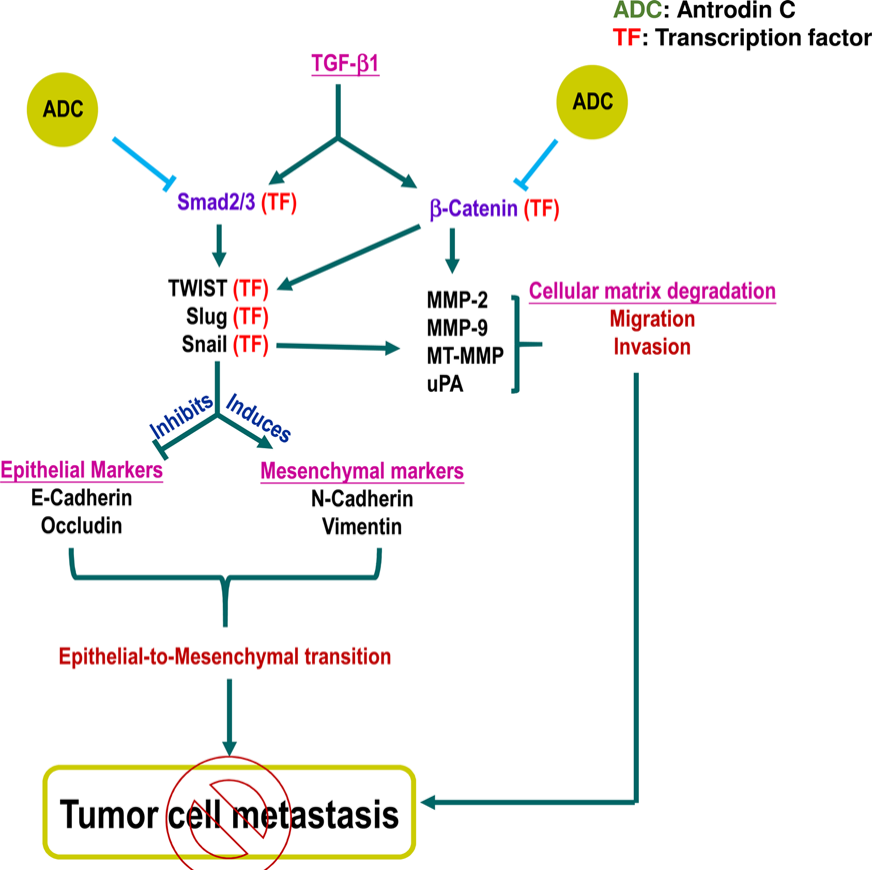
Schematic representation represents the anti-metastatic potential of ADC. TGF-β ligand binding with TGF-β receptor II (TGF-βRII) recruit TGF-βRI into a tetrameric receptor complex resulting in transphosphorylation and activation of TGF-βRI, which then phosphorylates Smad2 or Smad3. Phosphorylated Smad2/3 associate with Smad4 and translocate into the nucleus, where they activate transcription of target genes including snail, slug, and Twist. These genes regulate EMT through suppression of epithelial markers and induction of mesenchymal markers. Pretreatment with ADC inhibits TGF-β-induced EMT by inhibiting phosphorylation and transcriptional activation of Smad2/3. In addition, TGF-β activates β-catenin signaling cascade through the suppression of GSK3β, a negative regulator of β-catenin. The free form of β-catenin translocate into the nucleus and transcribe number of migration marker genes including MMP-2, MMP-9, and uPA. Pretreatment with ADC blocks TGF-β1-induced migration and invasion of MCF-7 cells through the suppression of transcriptional activation of β-catenin followed by down-regulation of MMP-2, MMP-9, and uPA. In addition, previous reports demonstrated that MMPs expression also regulated by snail, transcription factor regulates EMT. There is a possible that reduction in snail expression by may influence MMPs activity. Moreover, Twist was reported to be one of the down-stream target of β-catenin. Therefore, we believe that there may be a cross-talk existing between ADC-induced down-regulation of β-catenin and Twist.

*Antrodiacinnamomea* is a medicinal mushroom endemic to Taiwan and used for treating many diseases. In this study, we have examines the anti-metastatic effects of ADC, an active component in *Antrodiacinnamomea*. We found that ADC significantly blocked TGF-β1-induced migration and invasion of MCF-7 cells through the down-regulation of matrix-metalloproteinases (MMP-2, -9) via the suppression of transcription factor, β-catenin. In addition, ADC reversed TGF-β1-induced EMT, supporting by up-regulation of epithelial markers concurrently with down-regulation of mesenchymal markers, possibly through the modulation of transcriptional regulators Smad/Smad3. These pathways are summarized in Fig. 8.

EMT, a physiological process involved switching cell morphology from epithelial phenotype into mesenchymal phenotype, has been implicated to increase the aggressive ability of tumor cell metastasis [28]. Previous studies have demonstrate that TGF-β1, a pleiotropic factor regulating various cellular functions, induces morphological changes leading to more mesenchymal-like phenotypes. Consistent with others, our results confirmed that incubation of MCF-7 cells with TGF-β1 induced expression of N-cadherin and vimentin, mesenchymal markers, and suppress E-cadherin and occluding, epithelial markers. Interestingly, the pretreatment with ADC blockedTGF-β1-induced phenotypic changes by up-regulation of E-cadherin and occludin, and down-regulation of N-cadherin and vimentin in MCF-7 cells.

de Graauw et al have shown that treatment of MCF-7 cells with TGF-β1 induces cytoskeleton rearrangement in a complex formation of lamellipodia [29]. Changes in the cytoskeleton architecture especially filamentous-actin (F-actin) formation that regulates cell motility and cell adhesion, have been found to be essential for metastasis [30]. In line with this report, we also found that TGF-β1 induced a typical cytoskeleton rearrangement as evidenced by F-actin formation, whereas pretreatment with ADC blocked the process in MCF-7 cells.

One significant finding of this study is the demonstration of a regulatory role of ADC in Smad2/Smad3 phosphorylation. A canonical Smad2/Smad3-depedent pathway is activated and subsequently phosphorylated upon the binding of TGF-β1 ligand to TGF-β receptor. Phosphorylated Smad2/Smad3 complexes with Smad4, and then translocates to nucleus where EMT regulatory genes including Snail, Slug, TWIST, and Sip1 are transcribed [9,10], an important event has been noted in EMT for several cell types including breast and ovarian epithelial cells [31,32]. Here, our results confirmed that TGF-β1 indeed induced phosphorylation of Smad2 and Smad3 at Ser465/467 and Ser423/425 residues, subsequently, it translocated into the nucleus as evidenced by both Western blot analyses and immunostaining. Protein expression levels of Smad2/Smad3 downstream target genes such as Snail, Slug, and TWIST were increased upon TGF-β1 stimulation, and inhibited significantly by ADC treatments. These data suggest that ADC inhibited TGF-β1 inducible Smad2/Smad3 phosphorylation and transcriptional activation, leading to inhibition of Snail, Slug, and TWIST and up-regulation of E-cadherin and occludin in MCF-7 cells.

Another possible mechanism for ADC to inhibit metastasis could be regulation of extracellular matrix. Wnt/β-catenin, a canonical pathway is up-regulated by TGF-β. In unstimulated condition, β-catenin is constitutively degraded by a destructive complex comprising APC, axin, and GSK3β. However, upon stimulation with TGF-β1, GSK3β is send for proteasome degradation, which in turn promotes β-catenin nuclear translocation. The nuclear β-catenin binds to TCF/LEF transcription factors forming a heterotrimeric complex that activates β-catenin downstream target genes, including MMP-2, MMP-9, MT1-MMP, and uPA, which involve matrix degradation and cell migration [17–19]. In the present study, we also found that TGF-β1induced protein expression and activation of all MMP-2, MMP-9, uPA, and MT1-MMP, the activation was further inhibited by ADC in MCF-7 cells. In particular, MMP-2 activation requires proteolytic cleavage from its proform by MT1-MMP and, treatment with ADC had no effect on pro-MMP-2 level, whereas a significant inhibition was observed in cleaved-MMP-2 levels, suggesting that ADC inhibited cleavage of MMP-2 by down-regulation of MT1-MMP activity. Next, we confirmed that TGF-β1-induced nuclear translocation and transcriptional activity of β-catenin, a premise supported by the work of TGF-β1-enhanced GSK3β degradation and cytosolic β-catenin accumulation [33]. ADC inhibited GSK3β phosphorylation and increased steady state level of GSK3β in the cytoplasm, which subsequently enhanced proteasomal degradation of β-catenin. These results demonstrate that ADC inhibits MMPs and uPA activity in MCF-7 cells through suppression of β-catenin signaling cascade.

ADC is demonstrated to inhibit growth of Lewis lung carcinoma cells with an ED_50_ value of 7.5μg/mL or 22.7μM [23]. In this study we found that ADC has low cytotoxic towards MCF-7 cells (with ED_50_ values of 59.2 μM), a concentration varying to the ED_50_ value of 7.5μg/mL or 22.7μM reported in Lewis lung carcinoma cell system. To exclude the possibility that the anti-metastatic effect was due to its cytotoxicity, cells were incubated with curcumin (20 μM), a 2-fold stronger cytotoxic agent (curcumin ED_50_ value is 23.2 μM) than ADC (data not shown). This data support that ADC inhibiting MCF-7 cells metastasis is unlikely *via* its cytotoxic effects.

Results of the present study conclude that ADC inhibits TGF-β1 signaling *via* two inter-linked mechanisms in MCF-7 cells: (1) ADC inhibits TGF-β1-induced phosphorylation and nuclear translocation of Smad2/Smad3 followed by suppression of their transcriptional activation; and (2) ADC suppressed TGF-β1-induced transcriptional activation of β-catenin *via* inhibition of GSK3β activity. These various lines of evidence suggested that suggest that ADC could be a promising compound for developing chemotherapeutic/chemo-preventive agents targeting cancer metastasis.
